# Supplementary material for: Mitotic chromosome condensation requires phosphorylation of the centromeric protein KNL-2 in C. elegans
Source: J Cell Sci. 2021 Dec 2;134(23):jcs259088. doi: 10.1242/jcs.259088 (PMC8714079; doi:10.1242/jcs.259088)
Supplement: Supplementary information [file joces-134-259088-s1.pdf]

FIGURE S1

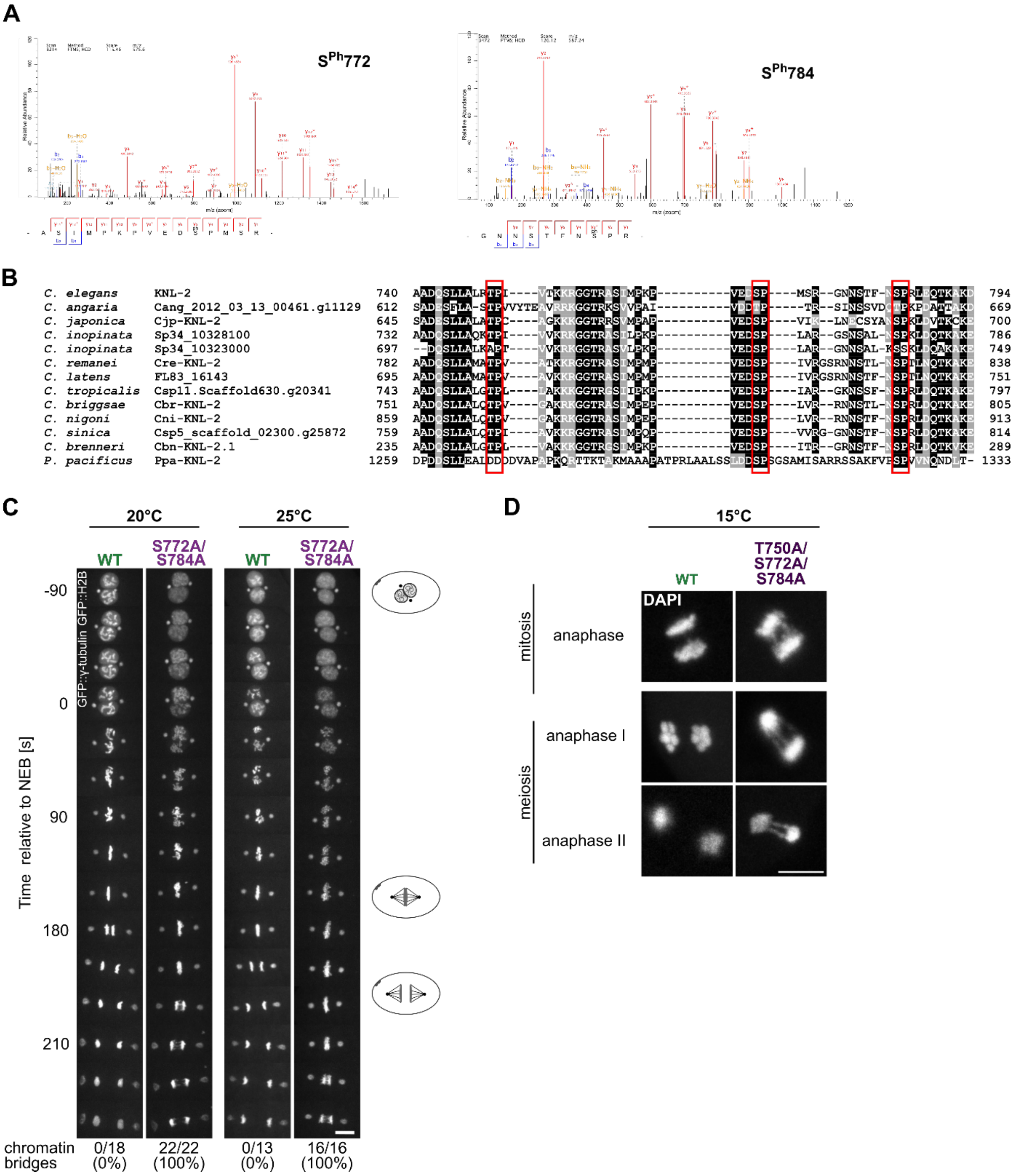

Fig. S1. KNLI 2 is phosphorylated on conserved residues T750, S772 and S784. (A) Exemplary spectra for peptides harbouring S772Ph or S784Ph modification identified by mass spectrometry. (B) Alignment of the C-terminal part of KNL-2 proteins from *Caenorhabditis* species, with KNL-2 from *Pristionchus pacificus* as an outgroup. The phosphosites identified in this study are marked with red boxes. (C) Kymographs illustrating the first embryonic division at 20°C and 25°C for wildtype (WT) and S772A/S784A embryos expressing GFP::H2B and GFP::γ-tubulin, with percentages of embryos exhibiting chromatin bridges during anaphase. NEB R nuclear envelope breakdown. Scale bar: 10 µm. (D) DAPI staining of fixed embryos from WT and T750A/S772A/S784A strains. Images show anaphases of mitotic and meiotic divisions at 15°C (permissive temperature for this strain). Scale bar: 5 µm.

Journal of Cell Science • Supplementary information

FIGURE S2

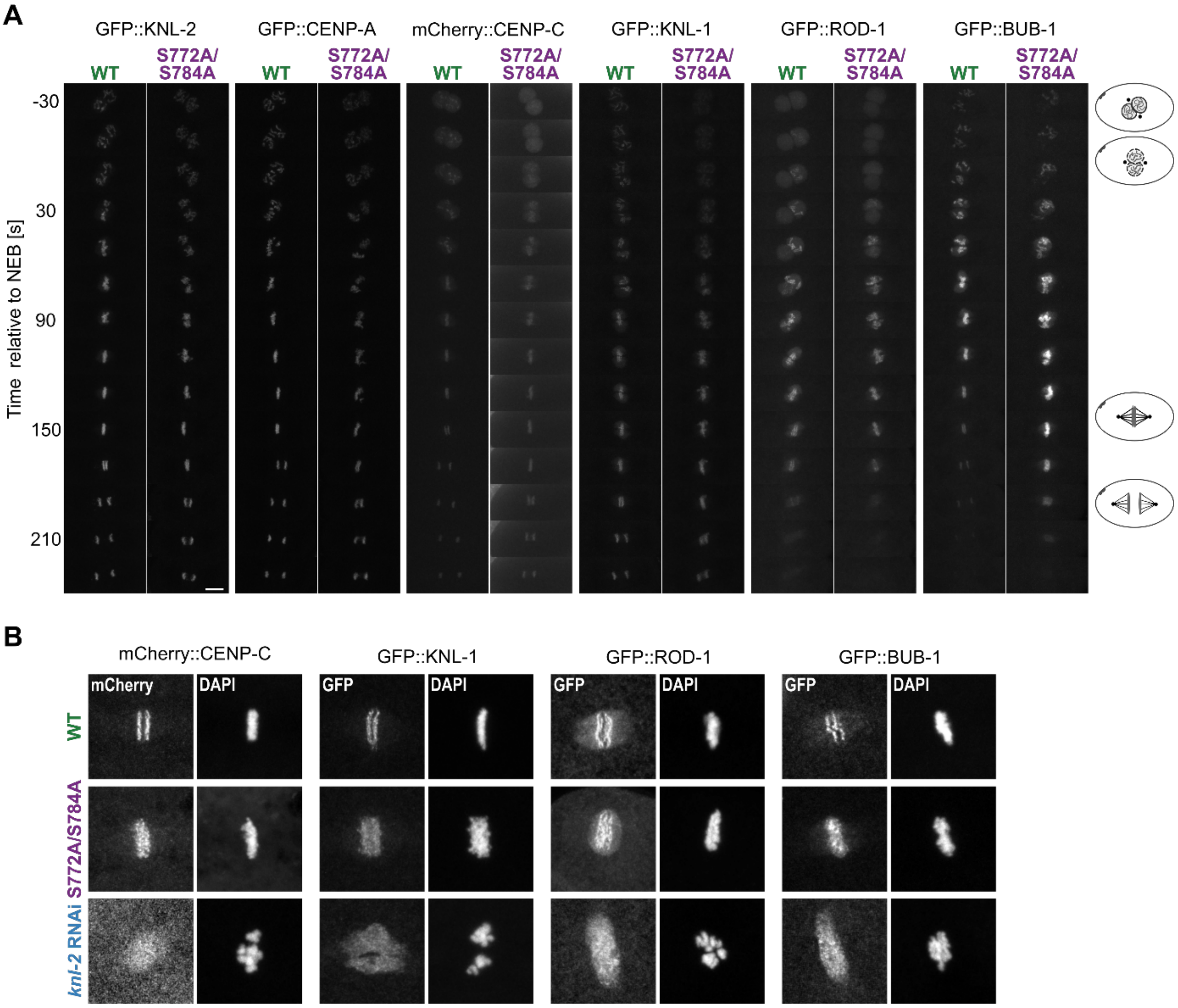

Fig. S2. Localisation of centromeric and kinetochore proteins is not affected by S772A and S784A KNL-2 mutations. (A) Kymographs showing the first embryonic division for wildtype (WT) and S772A/S784A embryos expressing the indicated GFP- or mCherry-tagged centromere and kinetochore proteins. NEB - nuclear envelope breakdown. Scale bar: 10 µm. (B) Images of fixed samples of embryos as in (A) and after *knl-2* RNAi, counterstained with DAPI. Scale bar: 5 µm.

FIGURE S3

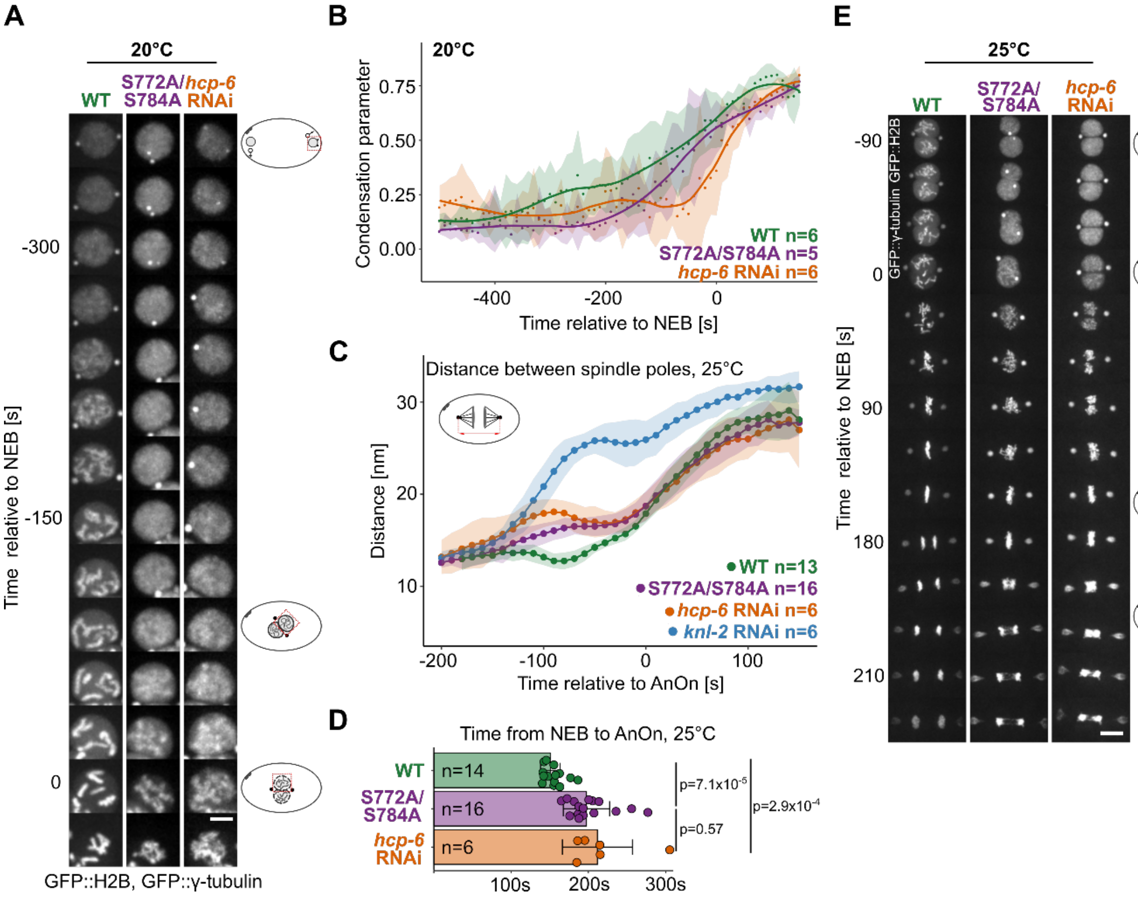

Fig. S3. Condensation impairment and resulting phenotypes in the S772A/S784A strain. Wild type (WT), S772A/S784A and *hcp-6* RNAi strains expressing GFP::H2B and GFP::γ-tubulin were analysed. (A) Kymographs of male pronuclei, illustrating the progression of chromosome condensation over time at 20°C (permissive temperature). Scale bar: 5 µm. (B) Quantification of the condensation parameter at 20°C for the time series in (A). Dots show the mean value of the condensation parameter for each timepoint, shaded areas represent s.d. Line plots were fitted with the R loess function (span=0.4) for illustrating the trend. n corresponds to the number of embryos scored per condition. The condensation parameter was calculated as described in detail in the Materials and Methods section. (C) Graph illustrating the changes of the distance between spindle poles in time. This graph also includes measurements from *knl-2* RNAi embryos for comparison. Dots show the average distance for each timepoint, shaded areas represent s.d., n denotes the number of scored embryos. AnOn - anaphase onset. (D) Quantification of the time between NEB and AnOn. Dots correspond to individual embryos scored (the total number n is indicated), error bars depict s.d. Statistical significance was assessed with Kruskal-Wallis test followed by Dunn's post hoc with Benjamini-Hochberg p-value adjustment. (E) Kymographs comparing the first embryonic division at 25°C. NEB - nuclear envelope breakdown. Scale bar: 10 µm.

FIGURE S4

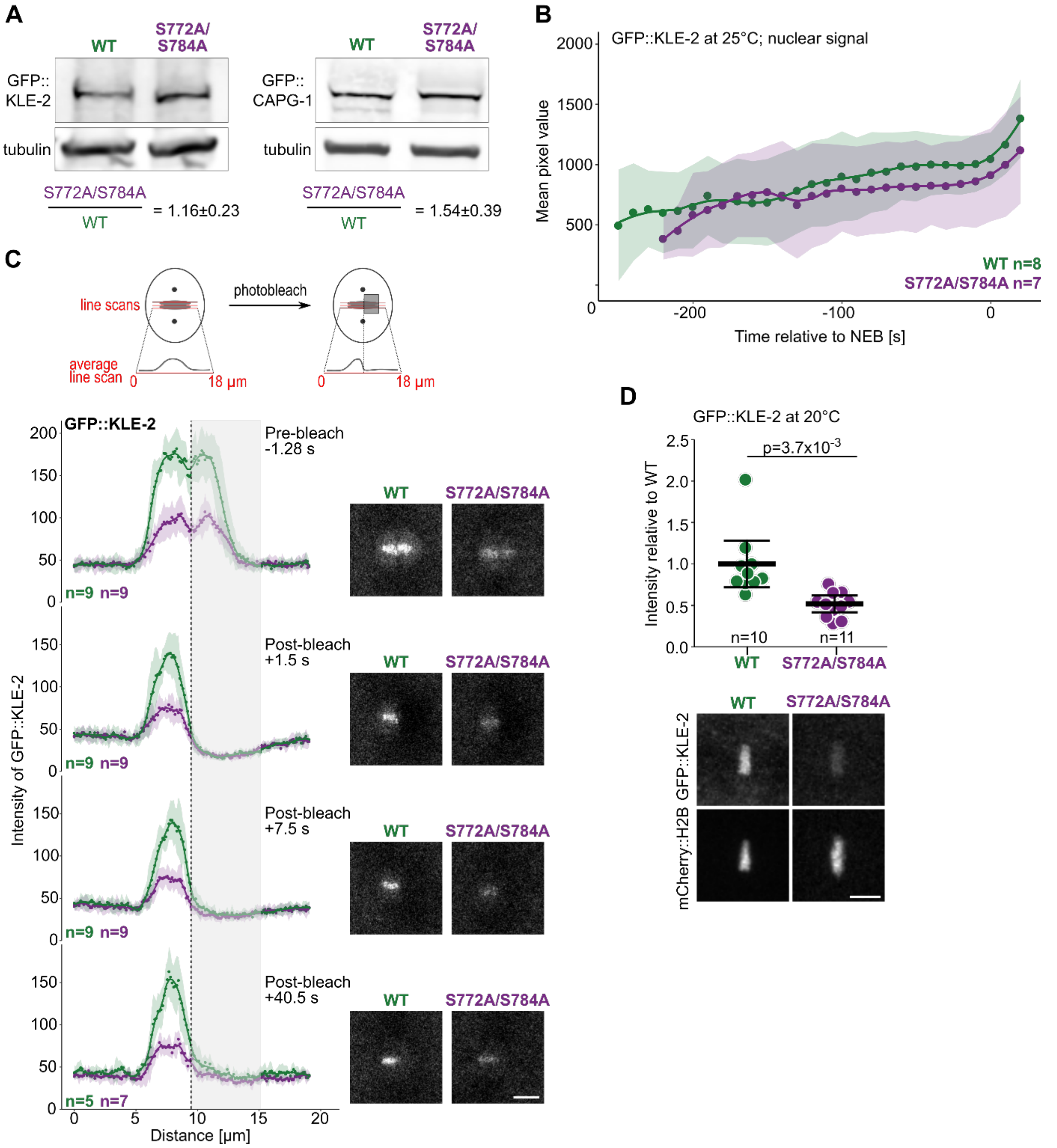

Fig. S4. Condensin complexes in the S772A/S784A mutant. (A) Western blot of total embryonic lysates from wild type (WT) or S772A/S784A strains. An antibody against GFP was used for detecting GFP::KLE-2 or GFP::CAPG-1, tubulin was used as a loading control. GFP band intensities were normalised to tubulin band intensities, and condensin subunit abundance in the S772A/S784A strain was compared to wildtype (average and s.d. from three experiments). (B) Quantification of the nuclear GFP::KLE-2 abundance in one cell embryos of wildtype and S772A/S784A strains. The line plot represents average value for all measurements, shaded areas represent s.d., n corresponds to the number of scored embryos per condition. (C) Top, a scheme of the FRAP experiment on the first embryonic metaphases with explanation of how the line graphs were generated. Left, line graphs illustrating the GFP::KLE-2 intensity distribution along the metaphase plate before and after the photobleach for WT and S772A/S784A embryos. Time points as indicated. Each dot represents the

average for the embryos quantified (n), shaded areas represent s.d., the lines were fitted with the R loess function (span=0.1) for illustrating the trend. The dotted line indicates the border between the non-bleached and photobleached parts of the metaphase plate, the grey shade indicates the bleached area. Right, representative images of the metaphases for indicated time points. Scale bar: 5 µm. (D) Quantification of GFP::KLE-2 signal on first embryonic metaphase plates at 20°C (permissive temperature). Each data point represents one scored embryo. t-test was used for testing the statistical significance. Representative images are shown below the quantifications. Scale bar: 5 µm.

Journal of Cell Science • Supplementary information

Table S1. Phosphopeptides identified in this study

Peptides	Modified residue no.	PSM	PEP	Localisation probability	Identified	Not phosphorylated peptide identified
ASIMPKPVEDS PMSR	772	5	0.000162664	0.995874	in 2 experiments	Yes
GNNSTFNSPR	784	2	0.000245025	0.999924	in 2 experiments	Yes
TPIVTK	*750*	*not identified in IP-MS experiments; T750 phosphorylated in in vitro assays*				
Searches performed with MaxQuant (Version 1.6.0.16)<br><br>PSM - Peptide Spectrum Match; PEP - Posterior Error Probability; Localisation probability - denotes how likely a given residue is modified						

Journal of Cell Science • Supplementary information

Table S2. List of strains used in this study

**Name**	**Genotype**	**Source**	**Comment**
N2	Wild type strain	CGC	used as parental strain for strain construction
EG6699	*ttTi5605 II; unc-119(ed3) III*	CGC	used as parental strain for strain construction
TH32	*ruIs32 [pie-1p::GFP::H2B + unc-119(+)] III; ddIs6 [tbg-1::GFP + unc-119(+)] V*	Oegema et al., 2001	expresses GFP::H2B and GFP::γ-tubulin, used for live imaging to inspect cell cycle progression (Fig. 1,3, S1, S3)
TG3828	*unc-119(ed3) III; gtIs3828[pie-1p:: gfp::kle-2 + unc-119(+)]; ltIs37 [pie-1p::mCherry::his-58 + unc-119 (+)] IV*	Sonneville et al., 2015	expresses GFP::KLE-2 and mCherry::H2B, used for live imaging and WB to track condensin II (Fig. 4, S4)
EKM36	*unc-119(ed3) III;cldIs5 [pie-1p::CAPG-1::GFP + unc-119 (+)]; ltIs37 [pie-1p::mCherry::his-58 + unc-119 (+)] IV*	Bembenek et al., 2013	expresses GFP::CAPG-1 and mCherry::H2B, used for live imaging and WB to track condensin I (Fig. 4, S4)
GCP529	*rod-1(lt62[gfp::rod-1]) IV; ltIs122[pAA64; pie-1p::mCherry::his-58 + cb-unc-119(+)]; unc-119(ed3) III*	Pereira et al., 2018	expresses GFP::ROD-1 and mCherry::H2B, used for live imaging to assess outer kinetochore behaviour (Fig. 2, S2)
TH229	*ddIs68 [bub-1::TY1::EGFP::3xFLAG(92C12) + unc-119(+)]*	Sarov et al., 2012	expresses GFP::BUB-1, used for live imaging to assess outer kinetochore behaviour (Fig. 2, S2)
TH243	*unc-119(ed3) III; ddIs153 [knl-1::TY1::EGFP::3xFLAG(92C12) + Cbr-unc-119(+)]*	Sarov et al., 2012	expresses GFP::KNL-1, used for live imaging to assess outer kinetochore behaviour (Fig. 2, S2)
FAS3	*ugeTi1[knl-2p::FLAG::GFP::knl-2 + cb-unc-119(+)] II; unc-119(ed3) III*	Prosée et al., 2020	expresses GFP::KNL-2, used for live imaging for checking KNL-2 levels (Fig. 2, S2)
FAS19	*hcp-3 (uge9[HA::hcp[illegible]3]) III*	Prosée et al., 2020	expresses HA::CENP-A, used as parental strain for other strain construction
FAS111	*knl-2(uge70[knl-2::2HA]) I;*	Prosée et al., 2020	expresses KNL-2::HA, used for IP-MS (Fig. 1, S1)

Journal of Cell Science • Supplementary information

FAS127	*knl-2 (uge88[knl-2::Ollas]) I; hcp-3 (uge9[HA::hcp[illegible]3]) III*	Prosée et al., 2020	expresses HA::CENP-A and KNL-2::Ollas, used as parental strain for other strain construction and as WT in staining experiments (Fig. 1, 2, 3, S1, S2)
FAS131	*hcp-3 (uge85[GFP::HA::hcp-3]) III*	Prosée et al., 2020	expresses GFP::CENP-A, used for live imaging for checking CENP-A levels (Fig. 2, S2)
FAS153	*hcp-4 (uge103[mCherry::FLAG::hcp-4]) I; knl-2 (uge88[knl-2::Ollas]) I; hcp-3 (uge9[HA::hcp-3]) III*	Prosée et al., 2020	expresses mCherry::CENP-C, HA::CENP-A and KNL-2::Ollas,used for live imaging for checking CENP-C levels (Fig. 2, S2)
FAS157	*knl-2(uge107[knl-2 T750A::Ollas]) I; hcp-3 (uge9[HA::hcp-3]) III*	This study	expresses HA::CENP-A and KNL-2::Ollas with T750A mutation (Fig. 1)
FAS217	*knl-2 (uge137[knl-2 S772A::Ollas]) I; hcp-3 (uge9[HA::hcp-3]) III*	This study	expresses HA::CENP-A and KNL-2::Ollas with S772A mutation (Fig. 1)
FAS218	*knl-2 (uge138[knl-2 S784A]) I; hcp-3 (uge9[HA::hcp-3]) III*	This study	expresses HA::CENP-A and KNL-2 with S784A mutation (Fig. 1), this strain has additional aa at KNL-2 C terminus (DYKDDDKR) from an unsuccessful FLAG-tagging attempt
FAS219	*knl-2 (uge139[knl-2 T750A S772A::Ollas]) I; hcp-3 (uge9[HA::hcp-3]) III*	This study	expresses HA::CENP-A and KNL-2::Ollas with T750A and S772A mutation (Fig. 1)
FAS156	*knl-2(uge106[knl-2 S772A S784A::Ollas]) I; hcp-3(uge9[HA::hcp-3]) III*	This study	expresses HA::CENP-A and KNL-2::Ollas with S772A and S784A mutations, used as parental strain for other strain construction and in staining experiments (Fig. 1, 2, 3, S2)
FAS220	*knl-2 (uge140[knl-2 T750A S772A S784A::Ollas]) I; hcp[illegible]3 (uge9[HA::hcp-3]) III*	This study	expresses HA::CENP-A and KNL-2::Ollas with S772A, S784A and T750A mutations (Fig. 1, S1)
FAS221	*knl-2(uge106[knl-2 S772A S784A::Ollas]) I; hcp-3 (uge85[GFP::HA::hcp-3]) III*	This study	expresses GFP::CENP-A and KNL-2::Ollas with S772A and S784A mutations, used for live imaging for checking CENP-A levels (Fig. 2, S2)

FAS222	*knl-2(uge106[knl-2 S772A S784A::Ollas]) I; hcp-4 (uge141[mCherry::FLAG::hcp-4]) I; hcp-3 (uge9[HA::hcp-3]) III*	This study	expresses mCherry::CENP-C, HA::CENP-A and KNL-2::Ollas with S772A and S784A mutations, used for live imaging for checking CENP-C levels (Fig. 2, S2)
FAS223 #	*knl-2(uge106[knl-2 S772A S784A::Ollas]) I; ugeTi142[knl-2p::FLAG::GFP::knl-2 S772A S784A + cb-unc-119(+)] II*	This study	expresses GFP::KNL-2 with S772A and S784A mutations, used for live imaging for checking KNL-2 levels (Fig. 2, S2)
FAS224 #	*knl-2(uge106[knl-2 S772A S784A::Ollas]) I; ruIs32 [pie-1p::GFP::H2B + unc-119(+)] III; ddIs6 [tbg-1::GFP + unc-119(+)] V*	This study	expresses GFP::H2B, GFP::γ-tubulin and KNL-2::Ollas with S772A and S784A mutations, used for live imaging to inspect cell cycle progression (Fig. 1, 3, S1, S3)
FAS225 #	*knl-2(uge106[knl-2 S772A S784A::Ollas]) I; gtIs3828[pie-1p:: gfp::kle-2 + unc-119(+)]; ltIs37 [pie-1p::mCherry::his-58 + unc-119 (+)] IV*	This study	expresses GFP::KLE-2, mCherry::H2B and KNL-2::Ollas with S772A and S784A mutations, used for live imaging and WB to track condensin II (Fig. 4, S4)
FAS226 #	*knl-2(uge106[knl-2 S772A S784A::Ollas]) I; cldIs5 [pie-1p::CAPG-1::GFP + unc-119 (+)]; ltIs37 [pie-1p::mCherry::his-58 + unc-119 (+)] IV*	This study	expresses GFP::CAPG-1, mCherry::H2B and KNL-2::Ollas with S772A and S784A mutations, used for live imaging and WB to track condensin I (Fig. 4, S4)
FAS230	*knl-2(uge106[knl-2 S772A S784A::Ollas]) I; rod-1(lt62[gfp::rod-1]) IV*	This study	expresses GFP::ROD-1 and KNL-2::Ollas with S772A and S784A mutations, used for live imaging to assess outer kinetochore behaviour (Fig. 2, S2)
FAS231 #	*knl-2(uge106[knl-2 S772A S784A::Ollas]) I; ddIs68 [bub-1::TY1::EGFP::3xFLAG(92C12) + unc-119(+)]*	This study	expresses GFP::BUB-1, and KNL-2::Ollas with S772A and S784A mutations used for live imaging to assess outer kinetochore behaviour (Fig. 2, S2)
FAS232 #	*knl-2(uge106[knl-2 S772A S784A::Ollas]) I; ddIs153 [knl-1::TY1::EGFP::3xFLAG(92C12) + Cbr-unc-119(+)]*	This study	expresses GFP::KNL-1, and KNL-2::Ollas with S772A and S784A mutations used for live imaging to assess outer kinetochore behaviour (Fig. 2, S2)

These strains may harbour the *119(ed3) III* mutation in homo- or heterozygous state, as they were obtained by genetic crossing and one of the parents was a *unc-119(ed3) III* homozygote.

Table S3. List of sgRNAs ,repair templates and expression vectors used in this study

oligo/ plasmid name	sequence	Description/Purpose
pFSa105	TCAATAATTGGGGTGTCAT	sgRNA sequence for CENP-A (HCP-3) N-terminal tagging
pFSa106	GCGATTTCCTCAATAATTG	sgRNA sequence for CENP-A (HCP-3) N-terminal tagging
oFSa0069	GTTTTCTAAATTTTATATTTT ATCAGGATAATCTTCGAACA ATGTACCCATACGATGTTCC TGACTATGCTGCAGCCTACC CATACGATGTTCCTGACTAT GCTGCAGATGACACACCAAT TATTGAGGAAATCGCCGAG CAAAATG	Repair template for CENP-A (HCP-3) N-terminal double HA tagging (contains PstI restriction site for screening)
pJW6	ATGCACAGAGATTAGTAGA	sgRNA sequence for KNL-2 C-terminal tagging; used also for modification of serines S772 and S784 (together with pJW29 and pJW30)
oJW0208	AGTAGAATAATTCCATGCAC AGAGATTACTTTCCCATGAG ACGTGGTCCGAGCTCGTTGG CGAATCCGGAGGATCCTCCC ATGTAGATAGATGTGTCTTC TTCACGC	Repair template for KNL-2 C-terminal Ollas tagging (contains BamHI restriction site for screening)
pJW1	ACAATCGTACTGCGGGTTCG	sgRNA sequence for CENP-C (HCP-4) N-terminal tagging
pJW2	GCAGTACGATTGTTCCTGG	sgRNA sequence for CENP-C (HCP-4) N-terminal tagging
pJW53	ATAGTTACAAAGAAGAGAG	sgRNA sequence for introducing KNL-2 T750A mutation
oJW0353	TGGCTGCTGATCAATCATTG CTCGCTCTCAGAgCtCCAATt GTcACtAAGAAGAGAGGAG GAACAAGAGC	Repair template for introducing KNL-2 T750A mutation (contains ScaI restriction site for screening)
pJW29	GTTTTACGCCTCTTGGCAA	sgRNA sequence for KNL-2 S772 and S784 modification
pJW30	ACAGAAGTTTTACGCCTCT	sgRNA sequence for KNL-2 S772 and S784 modification

pJW56		backbone: pCR-BluntII-TOPO (Invitrogen); contains: *knl-2* (2312-3024) with S772A and S784A mutations, AgeI and XhoI restriction sites for screening, and C-terminal Ollas tag
pJW57		backbone: pCR-BluntII-TOPO (Invitrogen); contains: *knl-2* (2312-3024) with S772A mutation, AgeI restriction site for screening, and C-terminal Ollas tag
pJW55		repair template for FAS223 construction; backbone: pCFJ151 (Addgene, Frøkjaer-Jensen et al., 2008); contains: *knl-2p* (2 kb upstream of ATG); 3xFLAG, GFP, *knl-2* cds with S772A and S784A mutations, *knl-2* 3'UTR (500 bp downstream of TAA)
pCGS143		vector for heterologous protein expression in *E. coli*; a derivative of pET42a (NOVAGEN; note that some vector features were changed); contains: T7 promoter, codon optimised *knl-2* cDNA sequence encoding residues 617-877; TEV cleavage site, GST, His-tag
pCGS147		vector for heterologous protein expression in *E. coli*; a derivative of pET42a (NOVAGEN; note that some vector features were changed); contains: T7 promoter, codon optimised *knl-2* cDNA sequence encoding residues 617-877 with T750A, S772A and S784A mutations; TEV cleavage site, GST, His-tag
pCGS151		vector for heterologous protein expression in *E. coli*; a derivative of pET42a (NOVAGEN; note that some vector features were changed); contains: T7 promoter, codon optimised *knl-2* cDNA sequence encoding residues 617-877 with S772A and S784A mutations; TEV cleavage site, GST, His-tag
pCGS155		vector for heterologous protein expression in *E. coli*; a derivative of pET42a (NOVAGEN; note that some vector features were changed); contains: T7 promoter, codon optimised *knl-2* cDNA sequence encoding residues 617-877 with T750A mutation; TEV cleavage site, GST, His-tag
pCGS159		vector for heterologous protein expression in *E. coli*; a derivative of pET42a (NOVAGEN; note that some vector features were changed); contains: T7 promoter, codon optimised *knl-2* cDNA sequence encoding residues 617-

		877 with S772A mutation; TEV cleavage site, GST, His-tag
pCGS160		vector for heterologous protein expression in *E. coli*; a derivative of pET42a (NOVAGEN; note that some vector features were changed); contains: T7 promoter, codon optimised *knl-2* cDNA sequence encoding residues 617-877 with S784A mutation; TEV cleavage site, GST, His-tag

Journal of Cell Science • Supplementary information

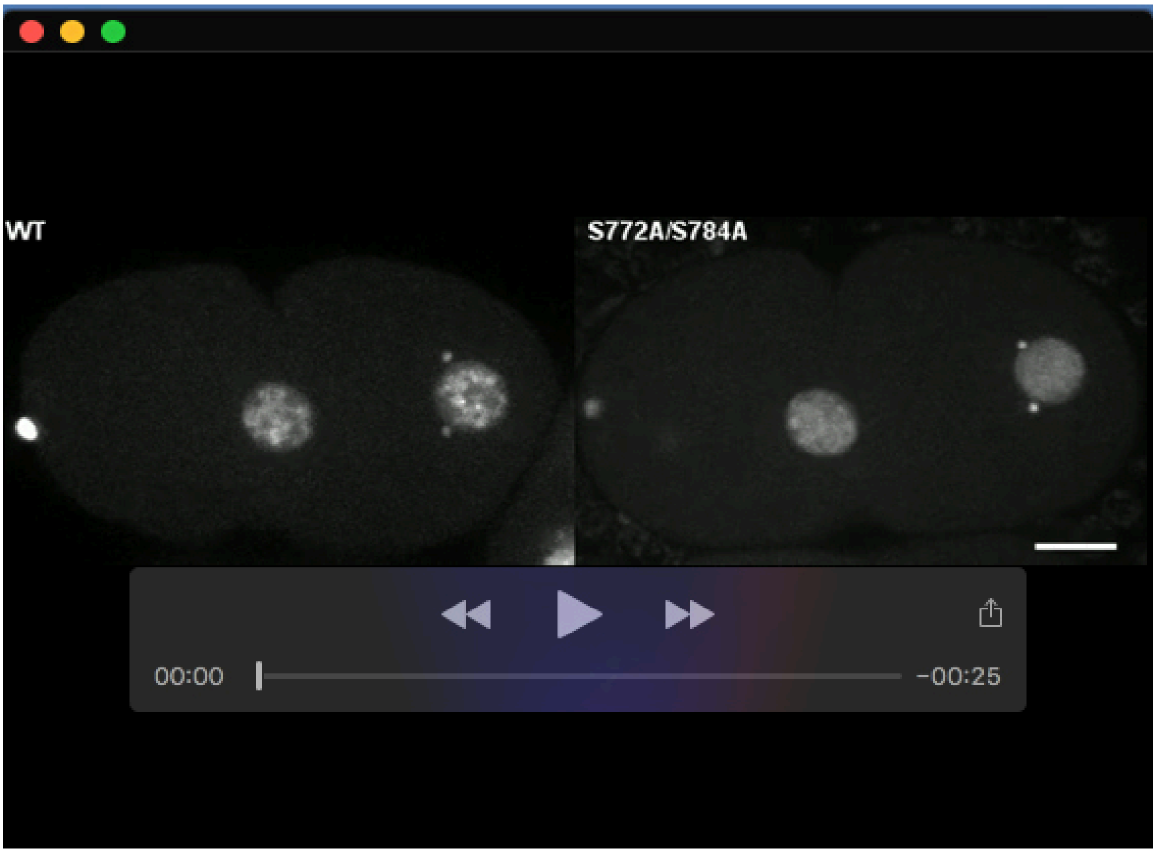

Movie 1. First embryonic cell division in WT and the S772A/S784A strain expressing GFP::H2B and GFP::γI tubulin at 20°C. Scale bar: 10 µm.

Journal of Cell Science • Supplementary information

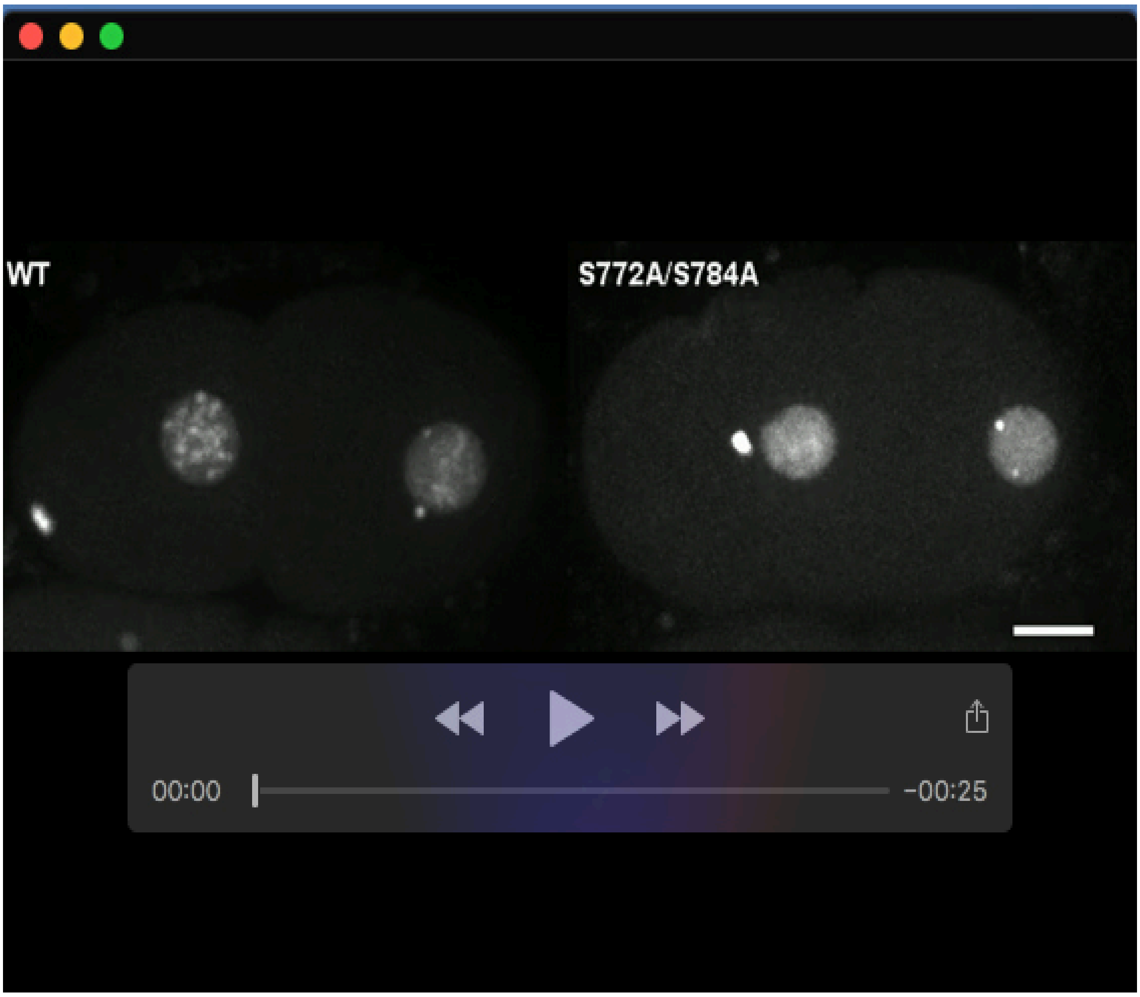

Movie 2. First embryonic cell division in WT and the S772A/S784A strain expressing GFP::H2B and GFP::γ-tubulin at 25°C. Scale bar: 10 µm.

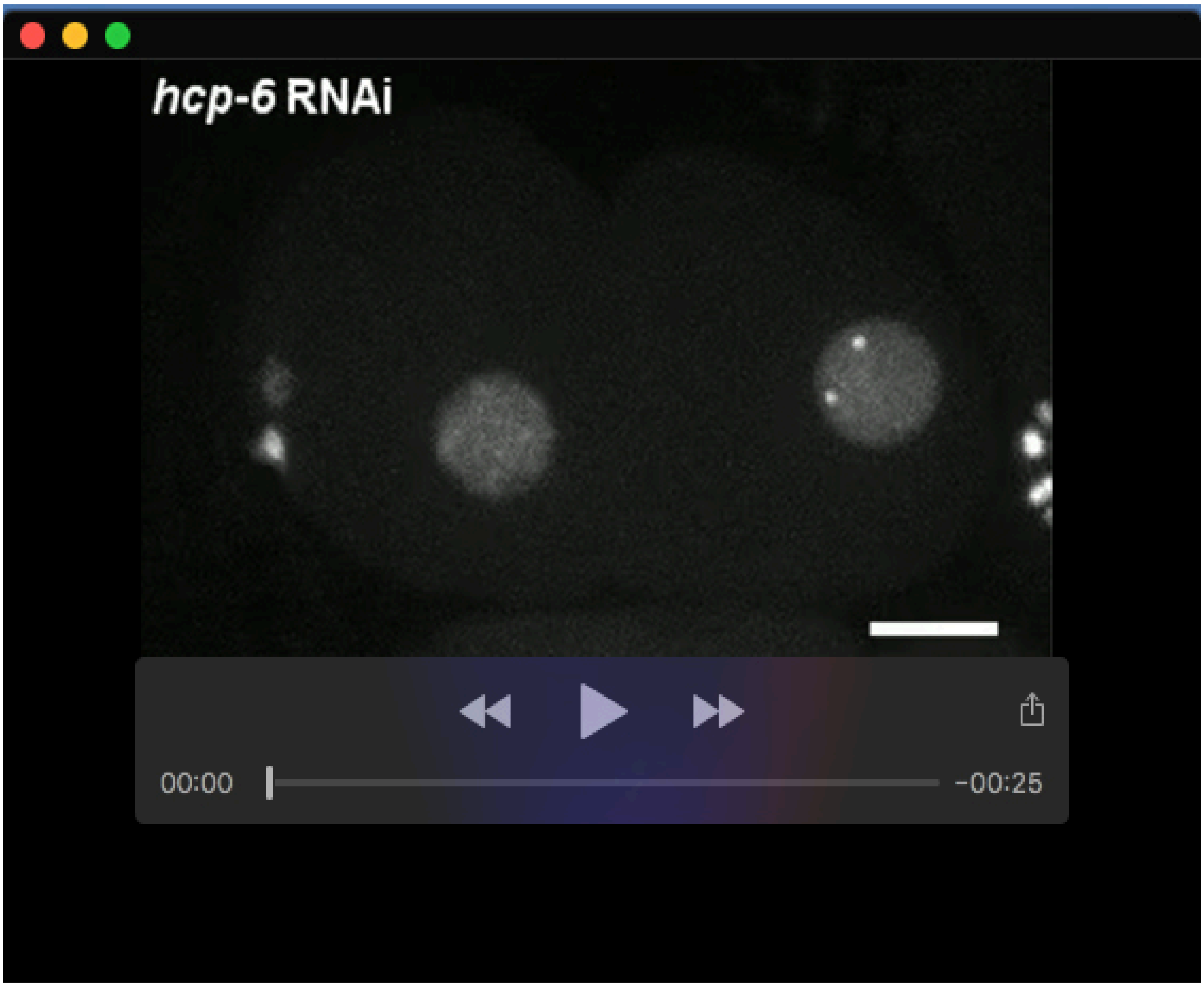

Movie 3. First embryonic cell division in a strain expressing GFP::H2B and GFP::γ-tubulin at 25°C after partial *hcp-6* depletion. Scale bar: 10 µm.

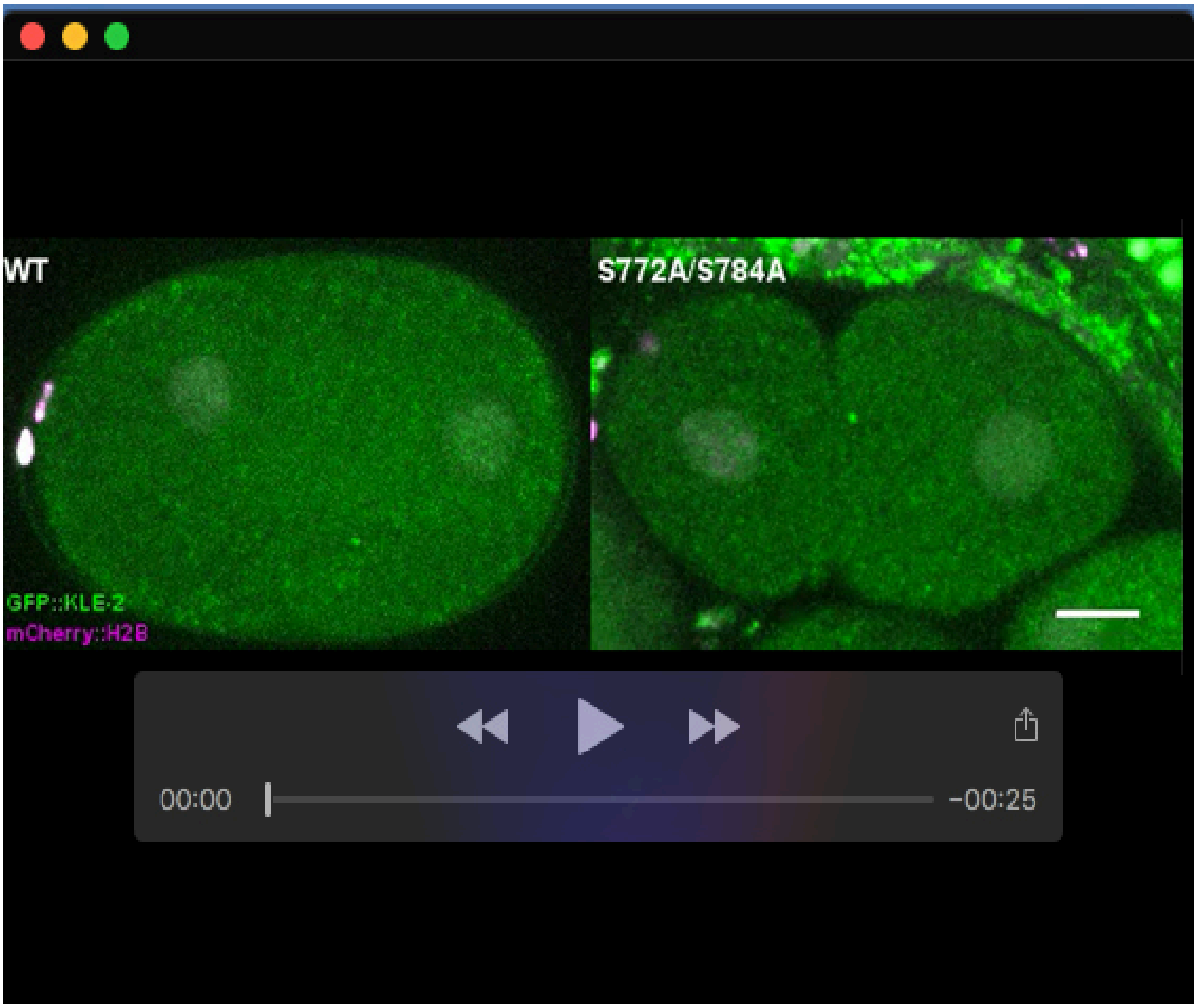

Movie 4. First embryonic cell division in WT and the S772A/S784A strain expressing GFP::KLEI-2 and mCherry::H2B at 25°C. Scale bar: 10 µm.

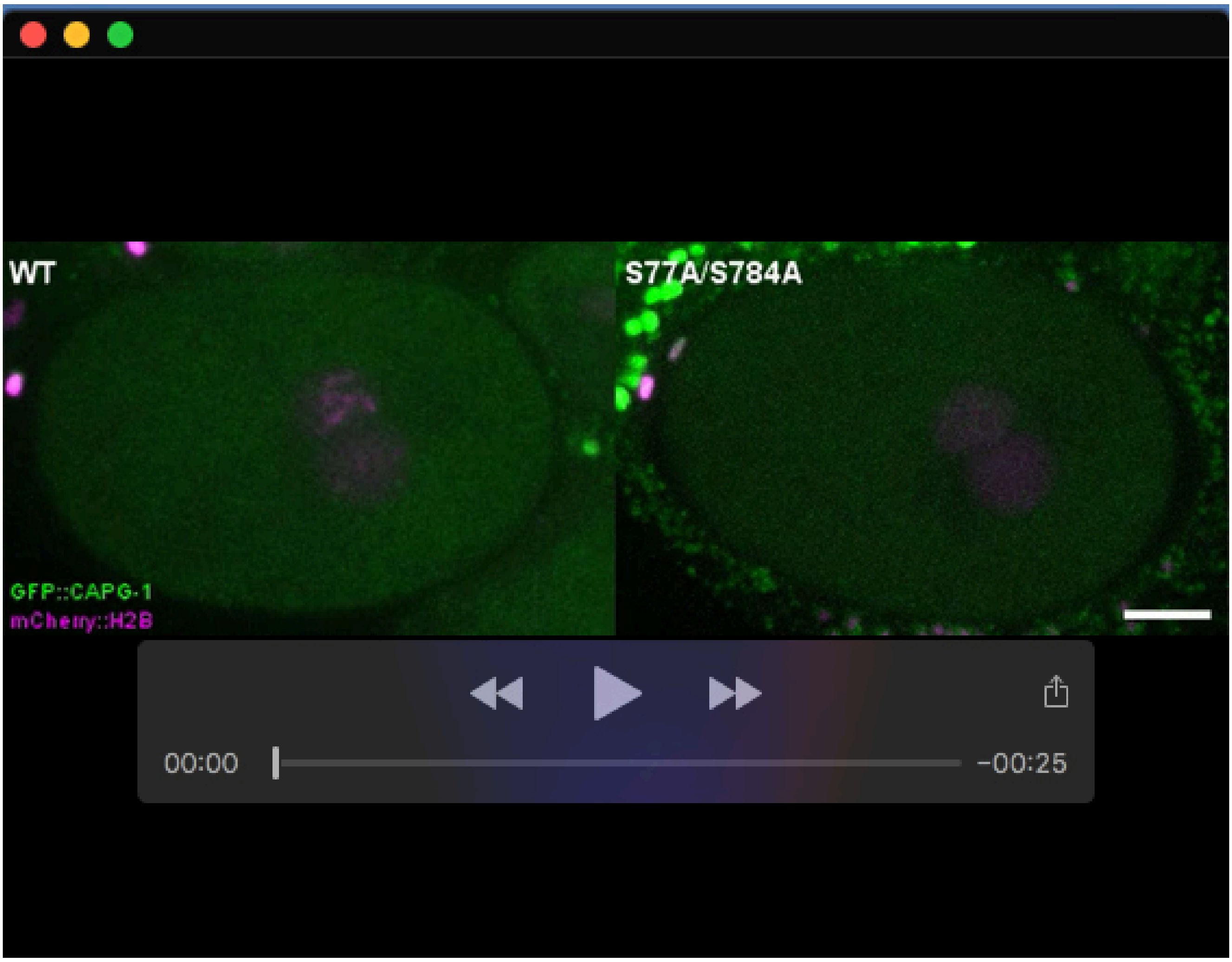

Movie 5. First embryonic cell division in WT and the S772A/S784A strain expressing GFP::CAPG-1 and mCherry::H2B at 25°C. Scale bar: 10 µm.

REFERENCES

Bembenek, J.N., Verbrugghe, K.J.C., Khanikar, J., Csankovszki, G., Chan, R.C., 2013. Condensin and the Spindle Midzone Prevent Cytokinesis Failure Induced by Chromatin Bridges in C. elegans Embryos. Current Biology 23, 937–946. https://doi.org/10.1016/j.cub.2013.04.028

Frøkjaer-Jensen, C., Davis, M.W., Hopkins, C.E., Newman, B.J., Thummel, J.M., Olesen, S.-P., Grunnet, M., Jorgensen, E.M., 2008. Single-copy insertion of transgenes in Caenorhabditis elegans. Nat. Genet. 40, 1375–1383. https://doi.org/10.1038/ng.248

Oegema, K., Desai, A., Rybina, S., Kirkham, M., Hyman, A.A., 2001. Functional Analysis of Kinetochore Assembly in Caenorhabditis elegans. J Cell Biol 153, 1209–1226. https://dx.doi.org/10.1083/jcb.153.6.1209

Pereira, C., Reis, R.M., Gama, J.B., Celestino, R., Cheerambathur, D.K., Carvalho, A.X., Gassmann, R., 2018. Self-Assembly of the RZZ Complex into Filaments Drives Kinetochore Expansion in the Absence of Microtubule Attachment. Current Biology 28, 3408-3421.e8. https://doi.org/10.1016/j.cub.2018.08.056

Prosée, R.F., Wenda, J.M., Özdemir, I., Gabus, C., Delaney, K., Schwager, F., Gotta, M., Steiner, F.A., 2021. Transgenerational inheritance of centromere identity requires the CENP-A N-terminal tail in the C. elegans maternal germ line. PLOS Biology 19, e3000968. https://doi.org/10.1371/journal.pbio.3000968

Sarov, M., Murray, J.I., Schanze, K., Pozniakovski, A., Niu, W., Angermann, K., Hasse, S., Rupprecht, M., Vinis, E., Tinney, M., Preston, E., Zinke, A., Enst, S., Teichgraber, T., Janette, J., Reis, K., Janosch, S., Schloissnig, S., Ejsmont, R.K., Slightam, C., Xu, X., Kim, S.K., Reinke, V., Stewart, A.F., Snyder, M., Waterston, R.H., Hyman, A.A., 2012. A Genome-Scale Resource for In Vivo Tag-Based Protein Function Exploration in C. elegans. Cell 150, 855–866. https://doi.org/10.1016/j.cell.2012.08.001

Sonneville, R., Craig, G., Labib, K., Gartner, A., Blow, J.J., 2015. Both Chromosome Decondensation and Condensation Are Dependent on DNA Replication in C. elegans Embryos. Cell Reports 12, 405–417. https://doi.org/10.1016/j.celrep.2015.06.046

Journal of Cell Science • Supplementary information
