# Supplementary material for: Mitotic chromosome condensation requires phosphorylation of the centromeric protein KNL-2 in C. elegans
Source: J Cell Sci. 2021 Dec 2;134(23):jcs259088. doi: 10.1242/jcs.259088 (PMC8714079; doi:10.1242/jcs.259088)
Supplement: Reviewer comments [file joces-134-259088_review_history.pdf]

Journal of Cell Science

Mitotic chromosome condensation requires phosphorylation of the centromeric protein KNL-2 in *C. elegans*

Joanna M Wenda, Reinier F Prosée, Caroline Gabus and Florian A Steiner
DOI: 10.1242/jcs.259088

Editor: David Glover

Review timeline
Original submission: 1 July 2021
Editorial decision: 4 August 2021
First revision received: 14 September 2021
Accepted: 25 October 2021

Original submission

First decision letter

MS ID#: JOCES/2021/259088

MS TITLE: Mitotic chromosome condensation requires phosphorylation of the centromeric protein KNL-2 in C. elegans

AUTHORS: Joanna M Wenda, Reinier F Prosée, Caroline Gabus, and Florian A Steiner
ARTICLE TYPE: Research Article

We have now reached a decision on the above manuscript.

To see the reviewers' reports and a copy of this decision letter, please go to: https://submit-jcs.biologists.org and click on the 'Manuscripts with Decisions' queue in the Author Area. (Corresponding author only has access to reviews.)

As you will see, the reviewers raise a number of substantial criticisms that prevent me from accepting the paper at this stage. They suggest, however, that a revised version might prove acceptable, if you can address their concerns. If you think that you can deal satisfactorily with the criticisms on revision, I would be pleased to see a revised manuscript. We would then return it to the reviewers.

We are aware that you may be experiencing disruption to the normal running of your lab that makes experimental revisions challenging. If it would be helpful, we encourage you to contact us to discuss your revision in greater detail. Please send us a point-by-point response indicating where you are able to address concerns raised (either experimentally or by changes to the text) and where you will not be able to do so within the normal timeframe of a revision. We will then provide further guidance. Please also note that we are happy to extend revision timeframes as necessary.

Please ensure that you clearly highlight all changes made in the revised manuscript. Please avoid using 'Tracked changes' in Word files as these are lost in PDF conversion.

I should be grateful if you would also provide a point-by-point response detailing how you have dealt with the points raised by the reviewers in the 'Response to Reviewers' box. Please attend to all of the reviewers' comments. If you do not agree with any of their criticisms or suggestions please explain clearly why this is so.

© 2021. Published by The Company of Biologists under the terms of the Creative Commons Attribution License (https://creativecommons.org/licenses/by/4.0/).

Reviewer 1

Advance summary and potential significance to field

This manuscript describes the requirement for phosphorylation of three residues in the C terminal tail of KNL-2 in regulating chromosome condensation in both meiosis and mitosis. The authors identify 2 in vivo phosphorylated residues in the C terminal tail of KNL-2, verify that these two and a third residue can be phosphorylated by CDK-1 in vitro and make mutations of these residues to analyze their in vivo consequence.

The authors find that these mutations are temperature sensitive, produce embryonic inviability and affect mitotic and meiotic chromosome segregation. Specifically, the authors observe chromosome bridging during anaphase. They report that these mutations are dispensable for CENP-A loading and kinetochore function but disrupt chromosome condensation, producing the defects in chromosomes segregation that they observe.

Additional experiments show that this defect in condensation can be attributed to a reduced recruitment of condensin II, which appears to be partially compensated for by an increased recruitment of condensin I. This paper is clearly written, beautifully presented and makes a significant contribution to the field, demonstrating that the role of KNL-2 in assembling kinetochores and regulating condensation can be mutationally uncoupled. In addition, the authors used control experiments (including hcp-6 mutants in their condensation assay, using knl-2 RNAi as a comparison for kinetochore protein recruitment, etc.) very effectively to tell their story. I had some minor concerns that should be addressed or clarified before publication.

Comments for the author

In the abstract the sentence “we show that KNL-2 is phosphorylated by CDK-1” should have in vitro added.
Line 148-149: “Since complete loss of KNL-2 function causes fully penetrant embryonic lethality, the KNL-2 T750A/S772A/S784A and S772A/S784A mutants exhibit a partial loss-of-function in addition to being thermosensitive.”
This is true of the S772A/S784A mutant but the T750A/S772A/S784A seems to be more like the KNL-2 complete loss of function, at least at 20 and 25 degrees. Perhaps the authors could change this to: “Since complete loss of KNL-2 function causes fully penetrant embryonic lethality at all temperatures, the KNL-2 T750A/S772A/S784A and S772A/S784A mutants exhibit a partial loss-of-function in addition to being thermosensitive.”
It would be helpful to have DAPI or the chromosomes labeled in some other way (GFP, mCherry) in Figure 2E particularly since there are some differences in staining (less clearly delineated kinetochores), for example with GFP::ROD-1.
Line 226: “Mitotic chromosomes started to form around 240s before NEB.” and “visible mitotic chromosome formation appeared only around 90s before NEB (Fig. 3A, B).” It might be useful to indicate these time points for control and mutant conditions on Figure 3A with an arrow or asterisk for the reader.

The condensation parameter graphs seems to be slightly modified from the original assay described in Maddox et. al. 2006. Can the authors provide some additional information about how they settled on the arbitrary value of .4 of max value for their assay in the Materials and Methods?
Line 258-259: “Early diakinetic nuclei typically contain well defined bivalents, but in the S772A/S784A strain individual chromosomes were hardly distinguishable at these stages (Fig. 3D) It would be helpful to mention -5 oocyte here. For example, (Fig. 3D, -5 oocyte)
Line 260-1: and in late diakinetic oocytes, six individual chromosome tetrads were visible in both strains (Fig. 3D).” Change chromosome tetrads to bivalents and mention -1 oocyte, similar to above
Were the meiosis experiments shown in Figure 3D performed at 25 degrees as well?
Lines 275-278: “This premature pole separation may be a consequence of delayed formation of attachments between kinetochores and microtubules, rather than of defects in kinetochore assembly, as we found that kinetochore proteins localise normally in the S772A/S784A strain (Fig. 2D, E).” Is premature pole separation observed in kinetochore null mutants? This information might be useful for readers here.

© 2021. Published by The Company of Biologists under the terms of the Creative Commons Attribution License (https://creativecommons.org/licenses/by/4.0/).

The authors should make more clear earlier in the text that the condensin localization and recruitment experiments in Figure 4 were also performed at 25 degrees.
The authors may want to discuss the the chromosome condensation phenotype in greater detail in their Discussion, specifically that the S772A/S784A mutant and hcp-6 mutant show similar, striking defects in condensation before NEB and then what appears to be a partial rescue that correlates with NEB and presumably, condensin I recruitment. This partial rescue would be entirely consistent with their observation that they observe more recruitment of condensin I at what appears to be a similar timepoint in S772A/S784A and hcp-6 mutants. It's not necessary for this paper, but what does the condensation parameter graph look like if condensin I is lost in their assay?

Reviewer 2

Advance summary and potential significance to field

Chromosome condensation and centromere/kinetochore assembly are two processes often seen as independent, that are critical for mitotic (and meiotic) fidelity.
Recent studies start to highlight how the two processes may be mechanistically interdependent. This is mostly evident in C. elegans, where the holocentric nature of the chromosomes makes this interaction potentially more pronounced. Defects in centromere core components have indeed been previously shown to result in chromosome compaction defects in this model organism. However, it has been difficult to establish whether these defects are an indirect consequence of perturbed centromeres or, instead, reflect a direct role of centromeric proteins in the establishment of proper chromosome architecture.

This interesting manuscript by Wenda and co-workers provides a major advance by uncovering a direct contribution of KNL2 in chromosome condensation, in addition to the well-established role in centromere maintenance. The described "separation of function" allele, that perturbs chromosome structure but not centromere maintenance is a critical tool to disentangle these different contributions.

Overall, the experiments are very well performed, the conclusions are solid and of great interest to chromosome biology field. I am therefore in favor of its publication. I have just a few issues that I would suggest be addressed before publication.

Comments for the author

1. The mass-spec data and the in vitro phosphorylation experiments, together with the consensus sequence underlying the identified sites, strongly suggest that these two residues are phosphorylated by cdk1. Yet, no experiment directly addresses if these residues are indeed phosphorylated by cdk1 in vivo. The authors should either directly test this in vivo (which admittedly may not be trivial considering cdk1 inhibition prevents mitotic entry) or tone down the inference of direct regulation. This is properly acknowledged in several parts of the manuscript (e.g. page 6, "likely mediated by CDK-1") but less so in other sections (e.g. abstract).

2. It is also not fully proven how KNL-2 phosphorylation promotes condensin II loading. Uncovering this at the mechanistic level may be beyond the scope of this paper. Yet, the authors provide interesting hypothesis (e.g. modulate its stability) that could be easily tested experimentally (e.g. FRAP studies).

3. Although I agree the nomenclature should follow the species-specific names, it would be useful to parallel the C elegans nomenclature with more "conventional" names of each protein studied. This is nicely performed for hcp-3/cenp-A and on page 10 for several condensin subunits, but not in other cases.
Having this correspondence at least the first time each gene is mentioned would make it easier to follow for a non-C. elegans reader.

4. It would be nice to describe a bit better the method used to quantify condensation rather than simply referring to the original paper. The method is indeed very nice, but the description of

© 2021. Published by The Company of Biologists under the terms of the Creative Commons Attribution License (https://creativecommons.org/licenses/by/4.0/).

“fraction of pixels below the threashold of 0.4 max value” without further explanation may sound a bit cryptic for a non-expert.

5. The discussion section nicely places the findings in the context of prior literature, particularly prior studies in c. elegans. Yet, it would be interesting to expand on what these results in a holocentric organism may mean for other species with monocentric chromosomes. Particularly as condensin II has been shown to be enriched at centromeres (as mentioned). Also, condensin I loading and His3Ser10 phosphorylation have been previously reported to start at the centromere (Hendzel et al. 1997; Oliveira et al 2007;) hence the “spreading” idea may be present beyond the referred yeast case. Overall, I found the discussion too centered in c elegans studies. A more open discussion would certainly increase the interest in this nice work.

6. Related with the previous point, it would be interesting to note potential differences between KNL-2 and Mis18. Particularly with the regard to its mitotic localization as Mis18 is only bound to chromosomes upon mitotic exit. Also, cdk1 inhibition is known to inhibit its activity. Hence, it’s unclear to me whether KNL-2 and M18BP1 are true functional orthologues. Are the identified phosphosites conserved in other species? Placing the findings in a broader context would be critical to increase the significance of this work with regard to evolutionary conservation of centromere maintenance pathways.

7. Figure 3D missing scale bar

First revision

Author response to reviewers' comments

We thank the reviewers and the editor for their positive assessment of our work, encouraging comments, and for the chance to submit a revised version of our manuscript. During the revision we have made changes to the text (especially in the Discussion section), revised some figure annotations for clarity (Fig 3, S3) and added additional data (S2B, S3C, S4C) that support our conclusions. The additions include:

- Snapshots of metaphase plates from fixed samples from embryos expressing the kinetochore components mCherry::CENP-C, GFP::KNL-1, GFP::ROD-1, GFP::BUB- 1, counterstained with DAPI were added as a new panel S2B.
- Quantification of spindle poles separation dynamics after *knl-2* RNAi (to illustrate a kinetochore-null-phenotype) was added to the existing graph in the panel S3C.
- Data from FRAP experiments on first embryonic metaphases in embryos expressing GFP::KLE-2 were added as a new panel S4C.

The changes are indicated by a blue font in the manuscript for easy reference.

Reviewer 1 Advance Summary and Potential Significance to Field:
This manuscript describes the requirement for phosphorylation of three residues in the C terminal tail of KNL-2 in regulating chromosome condensation in both meiosis and mitosis. The authors identify 2 in vivo phosphorylated residues in the C terminal tail of KNL-2, verify that these two and a third residue can be phosphorylated by CDK-1 in vitro and make mutations of these residues to analyze their in vivo consequence.
The authors find that these mutations are temperature sensitive, produce embryonic inviability and affect mitotic and meiotic chromosome segregation. Specifically, the authors observe chromosome bridging during anaphase. They report that these mutations are dispensable for CENP-A loading and kinetochore function but disrupt chromosome condensation, producing the defects in chromosomes segregation that they observe.
Additional experiments show that this defect in condensation can be attributed to a reduced recruitment of condensin II, which appears to be partially compensated for by an increased recruitment of condensin I. This paper is clearly written, beautifully presented and makes a significant contribution to the field, demonstrating that the role of KNL-2 in assembling kinetochores and regulating condensation can be mutationally uncoupled. In addition, the authors used control experiments (including hcp-6 mutants in their condensation assay, using

© 2021. Published by The Company of Biologists under the terms of the Creative Commons Attribution License (https://creativecommons.org/licenses/by/4.0/).

knl-2 RNAi as a comparison for kinetochore protein recruitment, etc.) very effectively to tell their story. I had some minor concerns that should be addressed or clarified before publication.

We thank the reviewer for their constructive comments. We appreciate their kind and encouraging feedback, and the recognition of our work's importance.

Reviewer 1 Comments for the Author:

1) In the abstract the sentence "we show that KNL-2 is phosphorylated by CDK-1" should have in vitro added.
This was also brought up in Reviewer 2 comment 1. We have added "in vitro" as suggested. (Line: 26)

2) Line 148-149: "Since complete loss of KNL-2 function causes fully penetrant embryonic lethality, the KNL-2 T750A/S772A/S784A and S772A/S784A mutants exhibit a partial loss- of-function in addition to being thermosensitive." This is true of the S772A/S784A mutant but the T750A/S772A/S784A seems to be more like the KNL-2 complete loss of function, at least at 20 and 25 degrees. Perhaps the authors could change this to: "Since complete loss of KNL-2 function causes fully penetrant embryonic lethality at all temperatures, the KNL-2 T750A/S772A/S784A and S772A/S784A mutants exhibit a partial loss-of-function in addition to being thermosensitive."
We have changed the sentence as suggested. (Lines: 148-149)

3) It would be helpful to have DAPI or the chromosomes labeled in some other way (GFP, mCherry) in Figure 2E, particularly since there are some differences in staining (less clearly delineated kinetochores), for example, with GFP::ROD-1.
We have added the requested DAPI images to label the chromosomes in a new Figure panel S2B. The less clearly delineated kinetochores are a consequence of incompletely bi- oriented chromosomes at metaphase in the S772A/S784A strain. This defect is indeed easiest to observe in case of the outer kinetochore staining, although it is visible also for GFP::CENP-A or GFP::KNL-2.

4) Line 226: "Mitotic chromosomes started to form around 240s before NEB." and "visible mitotic chromosome formation appeared only around 90s before NEB (Fig. 3A, B)." It might be useful to indicate these time points for control and mutant conditions on Figure 3A with an arrow or asterisk for the reader.
We have annotated the time points with arrows on Figure 3A and 3B, as requested.

5) The condensation parameter graphs seems to be slightly modified from the original assay described in Maddox et. al. 2006. Can the authors provide some additional information about how they settled on the arbitrary value of .4 of max value for their assay in the Materials and Methods?
This was also brought up by Reviewer 2 in comment 4. We now describe the procedure in more detail in the Materials and Methods section (lines: 641-660), including the information that several thresholds were tested and the one providing the most informative results was chosen. We followed the original assay form Maddox et al., 2006, where the authors also tested several threshold values to identify the one best describing the changes in pixel value distribution. The exact value of the optimal threshold will vary between different experimental settings (different microscopes, cameras, imaging settings etc.). To simplify the graphs, we replaced the long y-axis labels ("fraction of pixels below the threshold of 0.4 of the max value") with "Condensation parameter" and refer the readers to Materials and Methods for its definition.

6) Line 258-259: "Early diakinetic nuclei typically contain well defined bivalents, but in the S772A/S784A strain individual chromosomes were hardly distinguishable at these stages (Fig. 3D) It would be helpful to mention -5 oocyte here. For example, (Fig. 3D, -5 oocyte)
We have changed the text as suggested, and removed a repeated "observed" in the previous sentence. (Line: 258-261)

© 2021. Published by The Company of Biologists under the terms of the Creative Commons Attribution License (https://creativecommons.org/licenses/by/4.0/).

7) Line 260-1: and in late diakinetic oocytes, six individual chromosome tetrads were visible in both strains (Fig. 3D)." Change chromosome tetrads to bivalents and mention -1 oocyte, similar to above Were the meiosis experiments shown in Figure 3D performed at 25 degrees as well?
We have changed the text as suggested and added that the meiosis experiments in Fig. 3D were also performed at 25°C. (Lines: 256; 263-264)

8) Lines 275-278: "This premature pole separation may be a consequence of delayed formation of attachments between kinetochores and microtubules, rather than of defects in kinetochore assembly, as we found that kinetochore proteins localise normally in the S772A/S784A strain (Fig. 2D, E)." Is premature pole separation observed in kinetochore null mutants? This information might be useful for readers here.
We have added the pole-to-pole distance kinetics upon *knl-2* RNAi (Fig. S3C) to illustrate the kinetochore null phenotype. Indeed, knl mutants also show premature pole separation, but the kinetics of pole separation are very different from the ones observed in S772/7784A and hcp-6-depleted embryos. This result supports our interpretation that the delay in pole separation is caused by delayed MT attachments rather than a lack of kinetochore assembly. (Lines: 277-280)

9) The authors should make more clear earlier in the text that the condensin localization and recruitment experiments in Figure 4 were also performed at 25 degrees.
We now state at the beginning of the description of these experiments "We used GFP- tagged KLE-2 for visualising condensin II and GFP-tagged CAPG-1 for condensin I for comparison of condensin complex dynamics in wild type and S772A/S784A strains at 25°C.", and in the following paragraph also note that the GFP::KLE-2 and GFP::CAPG-1 levels were quantified at 25°C. (Lines: 301-302; 309-310)

10) The authors may want to discuss the the chromosome condensation phenotype in greater detail in their Discussion, specifically that the S772A/S784A mutant and hcp-6 mutant show similar, striking defects in condensation before NEB and then what appears to be a partial rescue that correlates with NEB and presumably, condensin I recruitment. This partial rescue would be entirely consistent with their observation that they observe more recruitment of condensin I at what appears to be a similar timepoint in S772A/S784A and hcp-6 mutants. It's not necessary for this paper, but what does the condensation parameter graph look like if condensin I is lost in their assay?
We expanded the discussion of the chromosome condensation defects to include the points suggested by the reviewer (lines: 409-413 and 419-420). We repeated our condensation parameter measurements for condensin I depletion (capg-1 RNAi) and observed that the dynamics of prophase condensation is not significantly different from the wild type. A previous study in *C. elegans* (Csankovszki et al., 2009) also reported lack of visible changes in prophase condensation after condensin I depletion or in condensin I mutants. We decided not to include the graphs in the manuscript, but we provide them alongside the response to reviewers.

NOTE: We have removed a figure which was provided for the referees in confidence.

Reviewer 2 Advance Summary and Potential Significance to Field:
Chromosome condensation and centromere/kinetochore assembly are two processes, often seen as independent, that are critical for mitotic (and meiotic) fidelity. Recent studies start to highlight how the two processes may be mechanistically interdependent. This is mostly evident in C. elegans, where the holocentric nature of the chromosomes makes this interaction potentially more pronounced. Defects in centromere core components have indeed been previously shown to result in chromosome compaction defects in this model organism. However, it has been difficult to establish whether these defects are an indirect consequence of perturbed centromeres or, instead, reflect a direct role of centromeric proteins in the establishment of proper chromosome architecture.
This interesting manuscript by Wenda and co-workers provides a major advance by uncovering a direct contribution of KNL2 in chromosome condensation, in addition to the well-established role in centromere maintenance. The described "separation of function" allele, that perturbs chromosome structure but not centromere maintenance is a critical tool to

© 2021. Published by The Company of Biologists under the terms of the Creative Commons Attribution License (https://creativecommons.org/licenses/by/4.0/).

disentangle these different contributions.

Overall, the experiments are very well performed, the conclusions are solid and of great interest to chromosome biology field. I am therefore in favor of its publication. I have just a few issues that I would suggest be addressed before publication.

We thank the reviewer for their constructive comments. We appreciate their kind and encouraging feedback, and the recognition of our work's importance.

Reviewer 2 Comments for the Author:

1. The mass-spec data and the in vitro phosphorylation experiments, together with the consensus sequence underlying the identified sites, strongly suggest that these two residues are phosphorylated by cdk1. Yet, no experiment directly addresses if these residues are indeed phosphorylated by cdk1 in vivo. The authors should either directly test this in vivo (which admittedly may not be trivial considering cdk1 inhibition prevents mitotic entry) or tone down the inference of direct regulation. This is properly acknowledged in several parts of the manuscript (e.g. page 6, "likely mediated by CDK-1") but less so in other sections (e.g. abstract).

A similar point was brought up in Reviewer 1 comment 1. We have added that CDK-1 phosphorylation was observed "in vitro" in the abstract and in the results. In the discussion, we have changed the text to "*likely* established by CDK-1". (Lines: 26; 134; 347)

2. It is also not fully proven how KNL-2 phosphorylation promotes condensin II loading. Uncovering this at the mechanistic level may be beyond the scope of this paper. Yet, the authors provide interesting hypothesis (e.g. modulate its stability) that could be easily tested experimentally (e.g. FRAP studies).
We have now added a condensin II FRAP experiment in Figure S4C. We photobleached half of the GFP::KLE-2 signal at metaphase in WT and S772A/S784A strains and monitored the signal changes in time. We did not observe recovery of the GFP::KLE-2 signal in the bleached area, indicating that the protein is not mobile at this stage of the cell cycle, as reported previously for condensin II in human cells (Gerlich et al., 2006). The lack of recovery was evident in both WT and S772A/S784A embryos, suggesting that the mobility/stability of condensin II binding is not majorly changed in the mutant strain. We therefore favour the hypothesis of less efficient GFP::KLE-2 recruitment in S772A/S784A embryos.
We note, however, that due to weak GFP::KLE-2 signal (relative to background) and very fast embryonic divisions in the *C. elegans* embryos, this experiment bears technical limitations. We could monitor the recovery only for a short time (until the anaphase onset). Our attempts to measure the recovery rates at earlier stages of the cell cycle, especially around prophase, when the condensin II complex is expected to be stably loaded onto the chromatin, failed due to technical limitations. Optimisation of FRAP would require genetic mutants to slow down the division and brighter fluorophores (or higher expression levels), which is beyond the scope of this study.
We have added the results to the manuscript and commented on them in the Discussion section. (Lines: 318-323; 404-406)

3. Although I agree the nomenclature should follow the species-specific names, it would be useful to parallel the C elegans nomenclature with more "conventional" names of each protein studied. This is nicely performed for hcp-3/cenp-A and on page 10 for several condensin subunits, but not in other cases. Having this correspondence at least the first time each gene is mentioned would make it easier to follow for a non-C. elegans reader.
We have added the missing information for HCP-4/CENP-C, KNL-1/KNL1, BUB-1/BUB1 and HCP-6/CAP-D3. (Lines: 202-203; 225)

4. It would be nice to describe a bit better the method used to quantify condensation rather than simply referring to the original paper. The method is indeed very nice, but the description of "fraction of pixels below the threashold of 0.4 max value" without further explanation may sound a bit cryptic for a non-expert.

© 2021. Published by The Company of Biologists under the terms of the Creative Commons Attribution License (https://creativecommons.org/licenses/by/4.0/).

This was also brought up by Reviewer 1 in comment 5. We have replaced the y-axis description of “fraction of pixels below the threshold of 0.4 of the max value” with “Condensation parameter” to simplify the graphs. In the figure legends, we refer the readers to the Materials and Methods section, where we now describe the procedure in more detail and define the “condensation parameter”. (Lines: 641-660)

5. The discussion section nicely places the findings in the context of prior literature, particularly prior studies in c. elegans. Yet, it would be interesting to expand on what these results in a holocentric organism may mean for other species with monocentric chromosomes. Particularly as condensin II has been shown to be enriched at centromeres (as mentioned). Also, condensin I loading and His3Ser10 phosphorylation have been previously reported to start at the centromere (Hendzel et al. 1997; Oliveira et al 2007;) hence the “spreading” idea may be present beyond the referred yeast case. Overall, I found the discussion too centered in c elegans studies. A more open discussion would certainly increase the interest in this nice work.
We have added references to the suggested papers in the discussion of the spreading of chromosome condensation originating at the centromere. (Lines: 376-384)
We agree that the discussion is mainly placing our findings in the context of previous C. elegans studies. We prefer to keep the scope relatively narrow in order to keep this part of our manuscript concise. Nevertheless, we hope that the additions made in response to comments 5 and 6, as well as to Reviewer 1 comment 10 satisfactorily address this criticism.

6. Related with the previous point, it would be interesting to note potential differences between KNL-2 and Mis18. Particularly with the regard to its mitotic localization as Mis18 is only bound to chromosomes upon mitotic exit. Also, cdk1 inhibition is known to inhibit its activity. Hence, it’s unclear to me whether KNL-2 and M18BP1 are true functional orthologues. Are the identified phosphosites conserved in other species? Placing the findings in a broader context would be critical to increase the significance of this work with regard to evolutionary conservation of centromere maintenance pathways.
Some of these points were already mentioned in the discussion of the initially submitted manuscript, but probably did not come across clearly enough. We have extended this part of the discussion to better highlight the differences between KNL-2 and M18BP1, and added the point about cell cycle timing of chromosome association. (Lines: 363-371)
We agree that with regard to chromosome condensation, KNL-2 and M18BP1 are likely not functional orthologues. With regard to their roles in CENP-A loading, they might fulfill similar roles, although the mechanistic details might also differ.

7. Figure 3D missing scale bar
We added missing scale bars to the pachytene and oocyte snapshots in the Figure 3D.

Second decision letter

MS ID#: JOCES/2021/259088

MS TITLE: Mitotic chromosome condensation requires phosphorylation of the centromeric protein KNL-2 in C. elegans

AUTHORS: Joanna M Wenda, Reinier F Prosée, Caroline Gabus, and Florian A Steiner
ARTICLE TYPE: Research Article

I am happy to tell you that your manuscript has been accepted for publication in Journal of Cell Science, pending standard ethics checks.

© 2021. Published by The Company of Biologists under the terms of the Creative Commons Attribution License (https://creativecommons.org/licenses/by/4.0/).

Reviewer 1

Advance summary and potential significance to field

This manuscript describes the requirement for phosphorylation of three residues in the C terminal tail of KNL-2 in regulating chromosome condensation in both meiosis and mitosis. The authors identify 2 in vivo phosphorylated residues in the C terminal tail of KNL-2, verify that these two and a third residue can be phosphorylated by CDK-1 in vitro and make mutations of these residues to analyze their in vivo consequence.
The authors find that these mutations are temperature sensitive, produce embryonic inviability and affect mitotic and meiotic chromosome segregation. Specifically, the authors observe chromosome bridging during anaphase. They report that these mutations are dispensable for CENP-A loading and kinetochore function but disrupt chromosome condensation, producing the defects in chromosomes segregation that they observe.

Additional experiments show that this defect in condensation can be attributed to a reduced recruitment of condensin II, which appears to be partially compensated for by an increased recruitment of condensin I. This paper is clearly written, beautifully presented and makes a significant contribution to the field, demonstrating that the role of KNL-2 in assembling kinetochores and regulating condensation can be mutationally uncoupled. In addition, the authors used control experiments (including hcp-6 mutants in their condensation assay, using knl-2 RNAi as a comparison for kinetochore protein recruitment, etc.) very effectively to tell their story and responded well to suggestions to improve their manuscript.
Comments for the author

I appreciate the changes and improvements that the authors have made to the manuscript and have no additional concerns. My only remaining suggestions are to assess the manuscript for typos before publication. I found two typos while going over the manuscript and there may be others:
line 280: “which is consistent the finding that kinetochore proteins localise normally” should be “which is consistent *with* the finding that kinetochore proteins localise normally”
Line 376: “Aside from C. elegans, a potential regulatory role of centromeres” C. elegans should be italicized

Reviewer 2

Advance summary and potential significance to field

In this revised version, the authors have addressed most of my comments in a satisfactory manner. I trust the manuscript is in a much better shape (particularly the discussion, that presents a better discussion on the potential functional divergence of mis18/knl-2). I am therefore favourable to the publication of this interesting work in JCS.

Comments for the author

In this revised version, the authors have addressed most of my comments in a satisfactory manner. I trust the manuscript is in a much better shape (particularly the discussion, that presents a better discussion on the potential functional divergence of mis18/knl-2). I am therefore favourable to the publication of this interesting work in JCS.

© 2021. Published by The Company of Biologists under the terms of the Creative Commons Attribution License (https://creativecommons.org/licenses/by/4.0/).
